# Gains in life expectancy from decreasing cardiovascular disease and cancer mortality – an analysis of 28 European countries 1995–2019

**DOI:** 10.1007/s10654-023-01039-8

**Published:** 2023-09-07

**Authors:** András Wéber, Mathieu Laversanne, Péter Nagy, István Kenessey, Isabelle Soerjomataram, Freddie Bray

**Affiliations:** 1 Cancer Surveillance Branch, International Agency for Research on Cancer, Lyon, France; 2 Hungarian National Cancer Registry and National Tumor Biology Laboratory, National Institute of Oncology, Budapest, Hungary; 3 Department of Molecular Immunology and Toxicology and the National Tumor Biology Laboratory, National Institute of Oncology, Budapest, Hungary; 4 Department of Anatomy and Histology, Laboratory of Redox Biology, University of Veterinary Medicine, Budapest, Hungary; 5 Chemistry Institute, University of Debrecen, Debrecen, Hungary; 6 Department of Pathology, Forensic and Insurance Medicine, Semmelweis University, Budapest, Hungary

**Keywords:** Life expectancy, Decomposition, Cancer causes of death, Cardiovascular mortality, Geographical visualization

## Abstract

**Background:**

Life expectancy (LE) is an indicator of societal progress among rapidly aging populations. In recent decades, the displacement of deaths from cardiovascular disease (CVD) and cancer have been key drivers in further extending LE on the continent, though improvements vary markedly by country, sex, and over time. This study provides a comparative overview of the age-specific contributions of CVD and cancer to increasing LE in the 27 European Union member states, plus the U.K.

**Methods:**

Cause-by-age decompositions of national changes in LE were conducted for the years 1995–1999 and 2015–2019 based on the standard approach of multiple decrement life tables to quantify the relative impact over time. The contributions of CVD and cancer mortality changes to differences in LE were computed by sex and age for each of the 28 countries. We examine the difference between the member states before 2004 (“founding countries”) and those which accessed the EU after 2004 (“A10 countries”).

**Results:**

Among men, declines in CVD mortality in the founding countries of the EU were larger contributors to increasing LE over the last decades than malignant neoplasms: 2.26 years were gained by CVD declines versus 1.07 years for cancer, with 2.23 and 0.84 years gained in A10 countries, respectively. Among women in founding countries, 1.81 and 0.54 additional life years were attributable to CVD and cancer mortality declines, respectively, while in A10 countries, the corresponding values were 2.33 and 0.37 years. Lung and stomach cancer in men, and breast cancer in women were key drivers of gains in LE due to cancer overall, though rising mortality rates from lung cancer diminished the potential impact of increasing female LE in both EU founding (e.g., France, Spain, and Sweden) and A10 countries (e.g., Croatia, Hungary, and Slovenia), notably among cohorts aged 55–70 years. Over the 25 years, the LE gap between the two sets of countries narrowed from 6.22 to 5.59 years in men, and from 4.03 to 3.12 years for women, with diminishing female mortality from CVD as a determinative contributor.

**Conclusion:**

This study underscores the continued existence of an East-West divide in life expectancy across the EU27 + 1, evident on benchmarking the founding vs. A10 countries. In EU founding countries, continuous economic growth alongside improved health care, health promotion and protection policies have contributed to steady declines in mortality from chronic diseases, leading to increases in life expectancy. In contrast, less favourable mortality trends in the EU A10 countries indicate greater economic and health care challenges, and a failure to implement effective health policies.

**Supplementary Information:**

The online version contains supplementary material available at 10.1007/s10654-023-01039-8.

## Introduction

Life expectancy (LE) at birth is an important measure of the general health and overall mortality at the population level within a given country [1]. Increasingly, it is also a focus of interest as an indicator of socioeconomic development and well-being that accounts for changing living conditions and environmental factors in rapidly aging societies [2]. In accordance with observed epidemiologic transitions [3], LE has been rising in many countries and world regions in the last decades [4], although the COVID-19 pandemic impacted negatively on LE in half of the member states of the European Union (EU) since 2020 [5].

Overall progress in extending life prospects is mostly related to the postponement of death at older ages from cardiovascular disease (CVD) and cancer, as the leading causes of death in Europe and worldwide [6]. There is however considerable diversity in the rates of decline in mortality across EU member states in CVD and cancer, with breakthroughs in the prevention and treatment of the former contrasting with more variable progress in the latter [7–9]. Indeed, an east-west divide is in evidence between the member states before 2004 (“founding nations”) and the countries which accessed the EU after 2004 (“A10”).

Several publications have illustrated aspects of geographic disparities in cancer-related mortality in Europe [10–12]. The driving force behind these deviations, as proposed by Meslé and Vallin [13, 14], and its practical applicability to past trends [15], relates to cycles of convergence and divergence in LE. The majority of the A10 countries experienced stagnating or decreasing life expectancies in the second half of the 20th century, in contrast to founding countries of the EU. The collapse of the socialist system laid the foundation of the epidemiological renewal for their populations [16], however, the effects of radical social transformation had an adverse impact on LE prospects as late as the mid-1990s. Thereafter, life expectancy began to rise again, which, together with the acceleration of globalization and integration raised the question as to whether the LE gap between the two distinct sets of transitioning countries was narrowing or widening [17]. As a response to this enquiry, this study aims to provide a comprehensive and timely overview of the age-specific contributions of CVD and cancer to increases in LE in the EU founding versus A10 countries.

## Data sources and methods

The total number of deaths from CVD and cancer (International Classification of Diseases 10th revision; ICD-10 I00-99, C00-99) by year of death, sex, and five-year age group for the period 1995–2019 were extracted from the Eurostat database for 28 countries (all EU member states and the U.K., denoted hereafter as EU27 + 1) [18]. The source of the underlying causes of deaths were provided by the national statistical institutes, based on death certificates, while corresponding lifetables by five-year age group and sex were obtained from the Human Mortality Database (HMD) [19]. For cancer deaths, the following disease groups were defined: all malignant neoplasms combined (ICD10 C00-99) alongside the most frequent type of deaths from cancer: stomach (C16), colorectum (C18-21), lung (C33-34), breast (C50), female genital organs (C51-58), prostate (C61), lymphoid, hematopoietic and related tissue (C81-96) and all residual cancers (C00-14, C15, C22-26, C30-32, C37-39, C40-41, C43-49, C60, C62-63, C64-68, C69-72, C73-75, C76-80, C97).

Cause-by-age decompositions of the changes in LE were computed for the years 1995–1999 vs. 2015–2019 using standard decomposition technique by Horiuchi et al. [20], based on the multiple decrement life tables provided by Preston et al. [21]. Averaging 5-year periods increased the robustness of our study and allowed us to specify more precisely the fundamental trends by age and cause of death responsible for changes in LE. We computed the cause-by-age contributions to the differences in LE in the 28 countries by sex. All calculations were executed in **R** [22] and visualizations created using the Geofacet package [23]. Further notes on specific LE calculations at the country levels are provided in Appendix A. The term *founding* is used to denote EU countries that joined the EU prior to 2004 (Austria, Belgium, Denmark, Finland, France, Germany, Greece, Ireland, Italy, Luxembourg, the Netherlands, Portugal, Spain, Sweden, and the U.K.) and *A10* those countries that were part of the 2004 enlargement of the EU (Bulgaria, Croatia, Czech Republic, Estonia, Hungary, Latvia, Lithuania, Poland, Slovakia, Slovenia).

## Results

Among men in the EU27 + 1, the median gains in LE were 5.25 years from 1995–1999 to 2015–2019, with declines in mortality rates from CVD contributing 2.26 years to LE compared with 1.02 years for cancer (Table 1). The corresponding LE increase in women was 3.87 years, with CVD and cancer mortality declines contributing 2.05 and 0.44 years to the gains in LE, respectively.

**Table 1:** Life expectancy at birth (years) and changes in the EU member states (plus the U.K.) due to mortality from cardiovascular disease and specific cancer types

A – men											
	Life expectancies at birth in years			Life expectancy changes by causes of deaths in years							
Country	1995–1999	2015–2019	difference	CVD	Cancer	Other cancers	Stomach	Colorectal	Lung	Prostate	Lymph. & Haemato.
Founding EU countries											
Austria	74.04	79.19	5.15	2.75	1.00	0.19	0.14	0.19	0.30	0.12	0.06
Belgium	74.03	79.03	5.00	1.81	1.45	0.59	0.07	0.12	0.46	0.14	0.06
Denmark	73.49	79.06	5.57	2.26	1.14	0.29	0.02	0.17	0.42	0.11	0.13
Finland	73.31	78.76	5.45	2.59	0.78	0.10	0.10	0.02	0.33	0.12	0.11
France	74.42	79.42	5.00	1.47	1.44	0.72	0.06	0.11	0.33	0.14	0.09
Germany	73.86	78.49	4.63	2.26	1.02	0.56	0.11	0.16	0.09	0.08	0.02
Greece	75.23	78.71	3.48	1.81	0.39	0.15	0.06	-0.04	0.14	0.04	0.03
Ireland	73.20	80.07	6.87	3.16	1.07	0.33	0.09	0.17	0.25	0.13	0.09
Italy	75.45	80.70	5.25	1.92	1.32	0.55	0.14	0.09	0.40	0.08	0.06
Luxembourg	73.60	79.79	6.19	2.34	1.57	0.81	0.10	0.14	0.37	0.10	0.07
Netherlands	74.99	80.07	5.08	2.39	1.17	0.69	0.11	0.08	0.17	0.10	0.02
Portugal	72.22	78.30	6.08	1.91	0.23	-0.06	0.16	0.01	0.00	0.10	0.02
Spain	75.10	80.34	5.24	1.59	1.10	0.44	0.13	0.03	0.32	0.11	0.08
Sweden	76.66	80.76	4.10	2.44	0.79	0.16	0.08	0.06	0.19	0.19	0.11
United Kingdom	74.46	79.28	4.82	2.70	0.98	0.18	0.11	0.12	0.41	0.08	0.08
**Total - median**	**74.04**	**79.28**	**5.15**	**2.26**	**1.07**	**0.33**	**0.10**	**0.11**	**0.32**	**0.11**	**0.07**
**A10 countries**											
Bulgaria	67.42	71.39	3.97	1.55	0.18	0.00	0.10	-0.01	0.07	-0.01	0.03
Croatia*	71.20	74.86	3.66	1.80	0.63	0.24	0.12	-0.01	0.24	0.00	0.04
Czech Republic	70.54	76.02	5.48	2.97	1.55	0.38	0.12	0.25	0.62	0.07	0.11
Estonia	63.95	73.65	9.70	3.43	0.79	0.17	0.18	0.02	0.38	-0.02	0.05
Hungary	66.28	72.62	6.34	2.16	1.05	0.41	0.12	0.03	0.38	0.04	0.07
Latvia	62.62	70.03	7.41	2.12	0.55	0.10	0.14	0.03	0.29	-0.04	0.04
Lithuania	65.08	70.33	5.25	1.33	0.48	-0.02	0.14	0.03	0.28	0.00	0.06
Poland	68.22	73.80	5.58	2.72	0.90	0.48	0.13	-0.02	0.29	-0.01	0.03
Slovakia	68.59	73.74	5.15	2.59	1.05	0.33	0.12	0.10	0.45	0.01	0.04
Slovenia	71.16	78.08	6.92	2.30	1.05	0.24	0.18	0.15	0.44	0.04	0.00
**Total - median**	**67.82**	**73.70**	**5.53**	**2.23**	**0.84**	**0.24**	**0.13**	**0.03**	**0.34**	**0.00**	**0.04**
**EU – median**	**73.31**	**78.71**	**5.25**	**2.26**	**1.02**	**0.29**	**0.12**	**0.08**	**0.32**	**0.08**	**0.06**
B – women											
	Life expectancies at birth in years			Life expectancy changes by causes of deaths in years							
Country	1995–1999	2015–2019	difference	CVD	Cancer	Other cancers	Colorectal	Lung	Breast	Fem. Gen. Organ.	Lymph. & Haemato.
Founding EU countries											
Austria	80.44	83.93	3.49	2.22	0.58	0.16	0.16	-0.13	0.19	0.13	0.06
Belgium	80.53	83.64	3.11	1.42	0.68	0.18	0.12	-0.09	0.30	0.12	0.05
Denmark	78.47	83.00	4.53	1.68	1.19	0.20	0.17	0.13	0.36	0.22	0.11
Finland	80.63	84.27	3.64	1.90	0.42	0.15	0.04	-0.07	0.14	0.05	0.11
France	82.24	85.41	3.17	1.10	0.39	0.16	0.08	-0.18	0.17	0.08	0.09
Germany	80.21	83.26	3.05	1.81	0.57	0.13	0.17	-0.02	0.17	0.11	0.01
Greece	80.71	83.86	3.15	2.15	0.23	0.22	0.02	-0.09	0.06	-0.01	0.03
Ireland	78.72	83.87	5.15	2.35	0.77	0.27	0.11	0.00	0.24	0.05	0.09
Italy	81.74	85.08	3.34	1.56	0.48	0.22	0.08	-0.07	0.15	0.04	0.06
Luxembourg	79.94	84.37	4.43	2.00	0.78	0.51	0.16	-0.15	0.12	0.11	0.03
Netherlands	80.46	83.29	2.83	1.52	0.40	-0.06	0.07	0.03	0.28	0.07	0.01
Portugal	79.52	84.32	4.8	2.14	0.41	0.17	0.05	-0.07	0.14	0.08	0.04
Spain	82.21	85.76	3.55	1.45	0.41	0.21	0.07	-0.16	0.17	0.05	0.07
Sweden	81.71	84.24	2.53	1.53	0.54	0.18	0.05	-0.05	0.17	0.10	0.10
United Kingdom	78.89	83.83	4.94	1.99	0.71	0.16	0.08	0.05	0.25	0.10	0.07
**Total – median**	**80.46**	**83.93**	**3.49**	**1.81**	**0.54**	**0.18**	**0.08**	**-0.07**	**0.17**	**0.08**	**0.06**
**A10 countries**											
Bulgaria	74.61	78.43	3.82	2.05	0.09	0.05	0.03	-0.06	0.03	0.01	0.02
Croatia*	78.38	81.05	2.67	1.97	0.14	0.16	-0.01	-0.11	0.06	0.02	0.02
Czech Republic	77.48	81.84	4.36	2.79	0.94	0.30	0.20	-0.03	0.21	0.18	0.08
Estonia	75.47	82.23	6.76	3.56	0.58	0.14	0.05	-0.02	0.16	0.18	0.07
Hungary	75.22	79.38	4.16	2.14	0.48	0.29	0.08	-0.18	0.14	0.09	0.06
Latvia	74.21	79.57	5.36	2.53	0.28	0.11	0.08	-0.01	0.07	0.01	0.02
Lithuania	76.15	80.31	4.16	2.14	0.37	0.10	0.05	-0.02	0.11	0.08	0.05
Poland	76.91	81.56	4.65	2.69	0.37	0.25	0.03	-0.07	0.04	0.10	0.02
Slovakia	76.7	80.57	3.87	2.73	0.35	0.17	0.08	-0.06	0.04	0.09	0.02
Slovenia	78.89	83.83	4.94	2.13	0.44	0.18	0.14	-0.18	0.19	0.11	0.01
**Total – median**	**76.43**	**80.81**	**4.26**	**2.33**	**0.37**	**0.17**	**0.07**	**-0.06**	**0.09**	**0.09**	**0.02**
**EU – median**	**78.89**	**83.64**	**3.87**	**2.05**	**0.44**	**0.17**	**0.08**	**-0.07**	**0.16**	**0.09**	**0.05**

Data source: HMD, Eurostat database.

* 2004–2009 vs. 2015–2019.

For men in founding EU countries, LE increased 5.15 years from 1995–1999 to 2015–2019, with 2.26 and 1.07 years gained due to decreasing mortality from CVD and cancer, respectively. The corresponding overall gains in the A10 countries of 5.53 years were of relatively greater magnitude, but the respective contribution of CVD and cancer was slightly lesser, at 2.23 and 0.84 years. Among women, LE increased 3.49 years in founding EU countries over the period, with 1.81 and 0.54 additional life years attributable to the respective declines in CVD and cancer mortality, whereas the gains were 4.26, 2.33 and 0.37 years in the A10 countries, respectively.

**Fig. 1:**
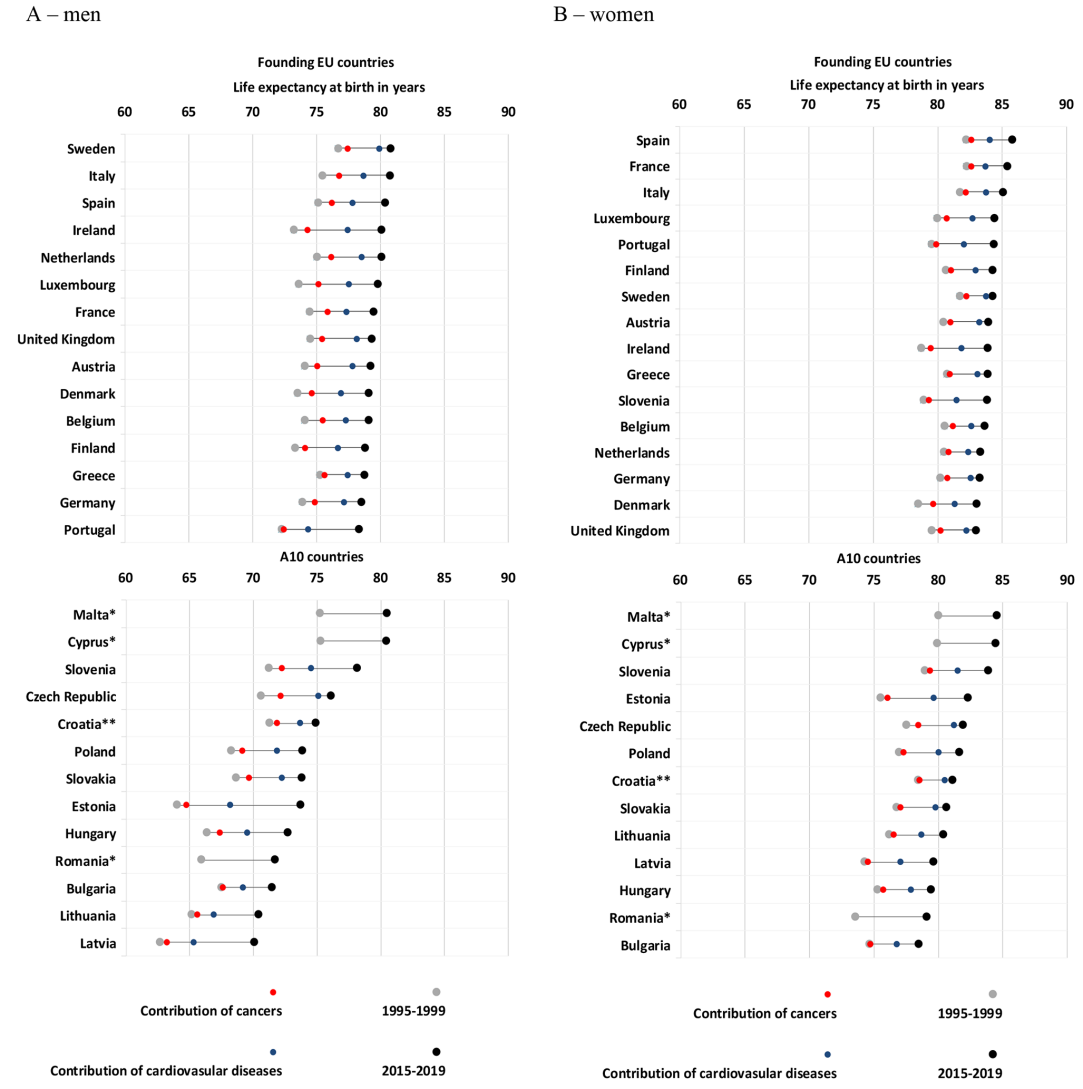
Life expectancy at birth in years and its change broken down into cancer and cardiovascular causes of death in the EU between 1995–1999 and 2015–2019 Data source: HMD, Eurostat. * Source: Eurostat database – mean average life expectancies for the corresponding periods. ** 2004–2009 vs. 2015–2019.

The East-West mortality gap in the EU27 + 1 appears to have closed only for CVD in female population, but not for cancer. Limited gains in LE seen among women in several founding EU countries (e.g., France: 0.39, Netherlands: 0.40, Spain: 0.41, Finland: 0.42 years) contrast with gains of around one year due to malignant neoplasms between 1995–1999 and 2015–2019 in Czechia and Denmark. The contribution of cancer to increasing LE were negligible for both sexes in Portugal, Bulgaria and Greece (Fig. 1; Table 1).

The decomposition of gains in LE enables an analysis of sex-specific differences according to period and cause of death by country. Historically there has tended to be larger gaps in overall LE in men and women in A10 compared with founding countries, as can be seen when comparing the Baltic countries (e.g. Latvia: 11.59, Estonia: 11.52, Lithuania: 11.07 years) vs. other Northern European countries (e.g. Sweden: 5.05, Denmark: 4.98, the U.K.: 4.43 years) in 1995–1999. The gender gap has narrowed only slightly in founding and A10 countries over the last 25 years, from 6.42 and 8.61 years in 1995–1999 to 4.65 and 7.12 years in 2015–2019, respectively. Considering the contribution of CVD mortality to LE in the A10 countries the sex-specific differences seems to be increasing (0.11 median years), whereas in founding nations, they are clearly diminishing (-0.45 median years). With respect to cancer, the sex-specific differences are decreasing everywhere, but to a slightly lesser extent in A10 countries (-0.47 median years) relative to founding countries (-0.53 median years), which in part relate to opposing lung cancer mortality trends by sex (Table 1).

**Fig. 2:**
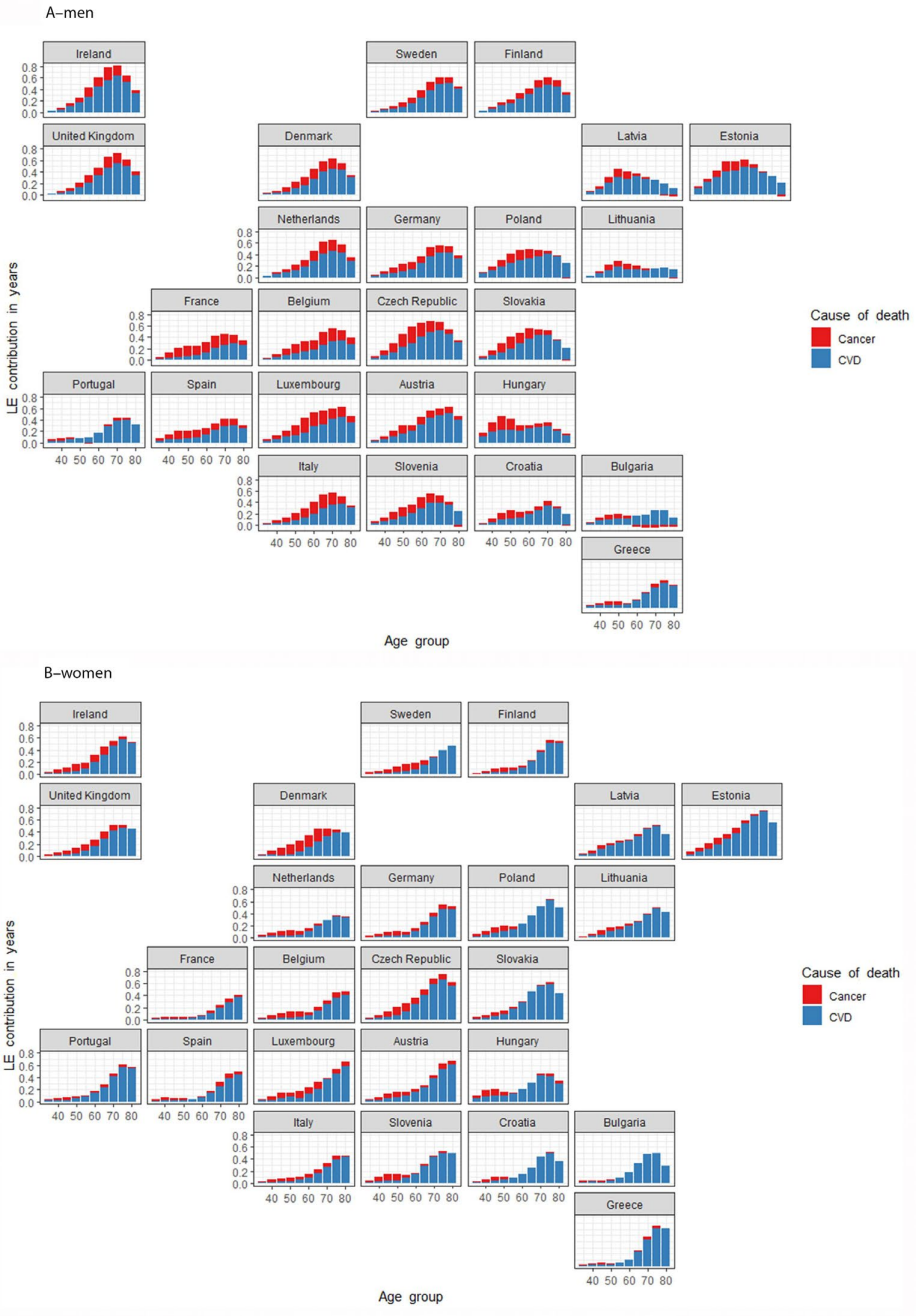
Map of age and cause of death specific contributions to life expectancy increase between 1995–1999 and 2015–2019 for the EU Data source: HMD, Eurostat database

With advancing age, an ever-increasing proportion of the mortality gains due to CVD determines the prospects of lengthening average life in a given country (Fig. 2). The contribution of cancer to LE is less emphatic but is more concentrated in younger age groups for both sexes. A strong negatively-acting cohort effect can be detected among older generations in most A10 countries, with the contributions to gains in LE for cancer restricted to ages less than 70 years for men, and ages less than 60 years for women.

**Fig. 3:**
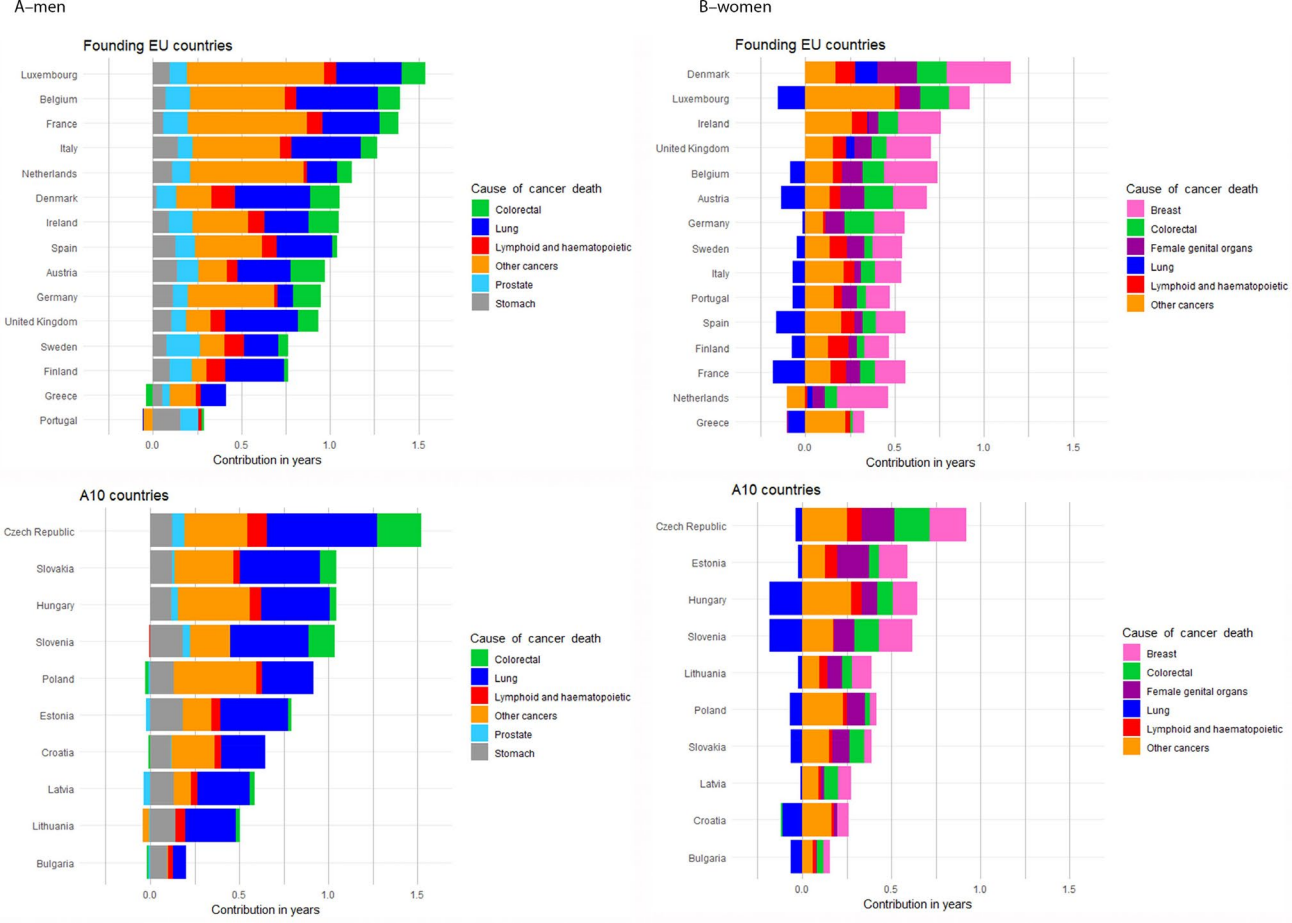
Cancer causes of death specific contributions to life expectancy increase between 1995–1999 and 2015–2019 for the EU Data source: HMD, Eurostat database

The gains in LE due to cancer among men in A10 countries largely result from declining lung cancer, and to a lesser extent, stomach cancer mortality rates, while gains in founding EU countries can be additionally attributed to diminishing mortality rates from colorectal and prostate cancer (Table 1; Fig. 3). Among women, gains in LE result from declines in colorectal and female genital cancer mortality can be observed in almost all EU27+1 countries, with more marked decreases of breast tumors related deaths in founding nations. However, these have diminishing impact given the increasing female lung cancer mortality rates in, for example, Hungary, Slovenia and Croatia; this offset of progress due to lung cancer’s rise is also seen in founding EU countries (e.g., in France, Spain and Austria) (Fig. 3). Mortality declines in other cancer types also substantially contributed to the increasing LE across the EU, though especially for men in founding countries. These included decreasing mortality trends in cancers of the lip, oral cavity, pharynx, larynx and bladder in men, and of kidney, liver and thyroid in women, though no discernible patterns emerge in founding or A10 countries. Denmark is an outlier in that, as with men, gains in LE among women result from declining mortality rates across the major cancer types. Mortality changes due to lymphoid and haematopoietic malignant neoplasms contributed minimally to increases in life expectancy.

**Fig. 4:**
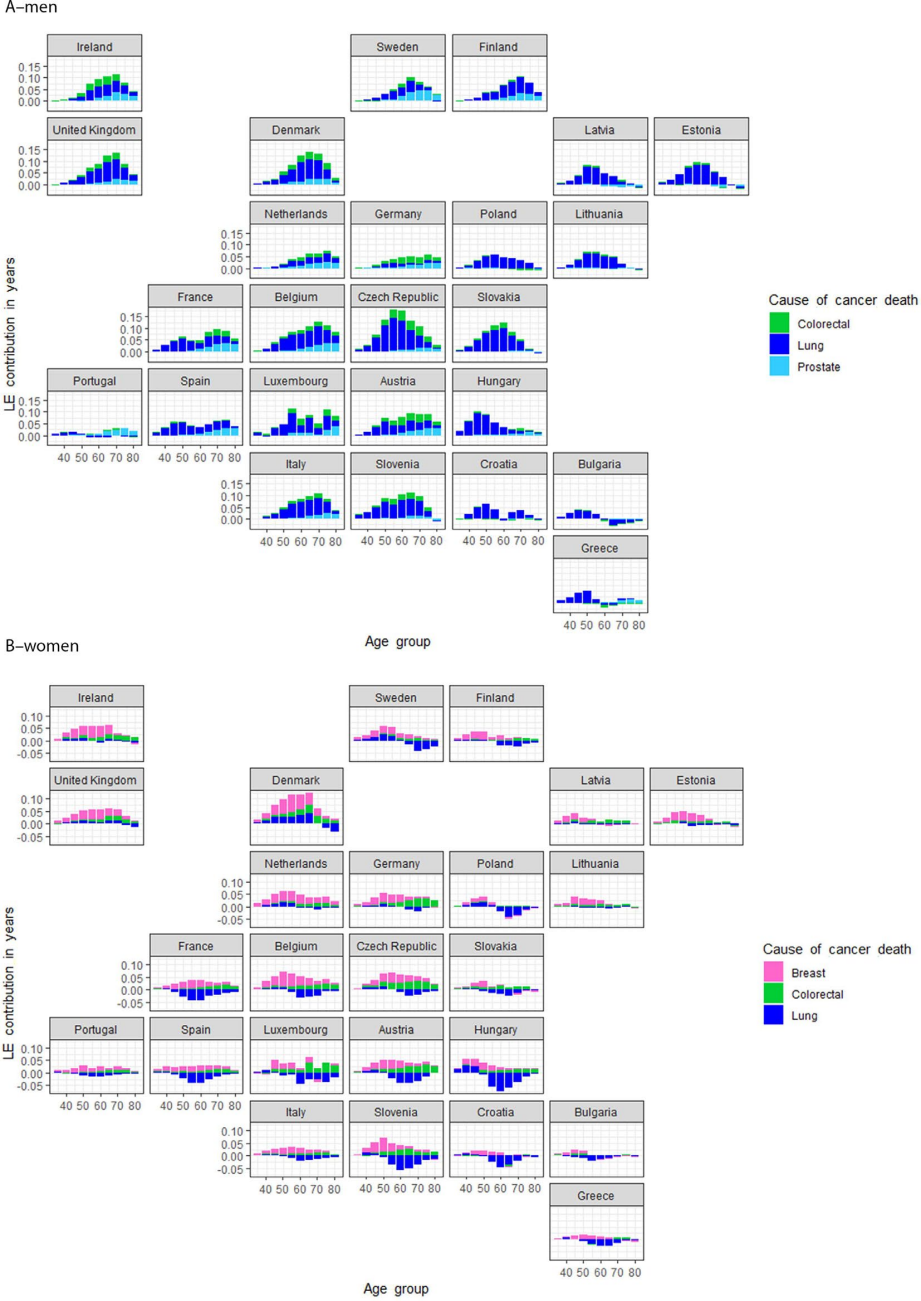
Map of age and cancer-specific causes of death specific contributions to increases in life expectancy between 1995–1999 and 2015–2019 for the EU Data source: HMD, Eurostat database

Figure 4 presents national variations in age-specific LE for the three cancers that contribute most by sex: colorectal, lung and prostate cancer in men, and breast, colorectal and lung cancer in women. LE gains in men are mainly due to lung cancer mortality declines across age groups. However, gains relate also to decreases in colorectal and prostate cancer mortality, through largely confined to older ages and the founding EU countries. Among women, breast cancer mortality declines also led to LE gains in founding countries, with gains in A10 countries tending to be lesser and mainly confined to younger ages. Where increasing mortality from lung cancer has impacted women most, e.g., in Sweden, France, Spain, Luxemburg, Slovenia, Austria, Hungary and Croatia, any gains in LE from declining breast cancer mortality rates have been more or less eliminated. An exception in the EU is Denmark where all three cancer types examined contributed to increasing LE in women, but only up to 65 years.

## Discussion

In the founding EU countries, continuous economic growth alongside improved health care (e.g. detection and treatment of hypertension, cancer screening and improved treatment) and health promotion and protection policies (e.g. tobacco control and improved road safety) have contributed to steady declines in the mortality of chronic diseases and subsequent increases in life expectancy. In contrast, the less favourable mortality trends in the A10 countries indicate greater economic and health-care challenges and a failure to implement effective health policies [24]. The root causes for such mortality trends include adverse social determinants of health under the backdrop of interrupted economic development, the so-called “state socialist mortality syndrome”, and the transition from socialist to capitalist systems [25–27]. At the micro level, there have been low levels of health awareness, that have manifested, for example, in high levels of alcohol and tobacco consumption, mostly among lower socioeconomic groups, whereas at the macro level, socialist economies in crisis have been unable to generate the necessary resources for modern and expensive medical technologies [16].

The negative consequences on cancer and CVD mortality are evident in this study of A10 versus founding EU countries. While decreasing inequalities in life expectancy can be observed throughout the EU27 + 1 countries over the quarter century, the absolute level of national LE, and the differences between the two regions are largely maintained, with LE higher and recent gains more evident in the founding EU countries. These countries have observed steep declines in cardiovascular mortality, as part of the so-called “cardiovascular revolution” due to major advances in the control of cardiovascular risk factors (smoking, blood pressure, and cholesterol levels) and improvements in medical care [28] that began in the late-1960s. These occurred decades earlier in founding EU vs A10 countries [10] and advances continues to this day, as can be seen in terms of declining mortality from ischemic heart disease [29] and acute myocardial infarction [30]. However, recent studies have also shown such reductions in CVD mortality to be waning in some high-income settings [31].

Progress against cancer and the extent to which different strategies across the cancer control continuum serve to control the disease has been deliberated for close to 50 years [32]. The assessment of cancer mortality trends and their impact on LE are necessarily complex given malignant neoplasms embrace over 200 types, each with differing (known or unknown) determinants for which selected primary, secondary, and tertiary interventions may be effective in controlling the disease. The continuous decreases in cancer mortality suggest founding EU countries are undergoing cancer transitions not dissimilar to those hypothesized in Japan [33], with a shift away from cancers triggered by unhealthy lifestyles (e.g.: smoking and drinking) and infection (stomach and cervix malignant neoplasms) towards a cancer profile more associated with affluence and non-infectious causes. Yet A10 countries also appear to be in a recovery phase and a transition from the epidemiological crisis to socioeconomic advancement made possible, in some countries, by regime change that has been accelerated with entry to the EU in 2004. Indeed, CVD mortality started to fall steeply from the early-2000s in these countries, though corresponding progress in cancer was confined to lung and stomach cancer through tobacco control [34] and the “unplanned triumphs” of primary prevention, respectively [35]. All of these resulted in disruptions of the life prospects gains in post-socialist societies in recent decades, drawing attention to the essential and persisting social inequalities in cancer mortality [36].

Tobacco consumption is a key risk factor in the development of CVD [37] and numerous cancer types [38]. Recent studies highlight that the excess risk of CVD is rapidly reversible through tobacco cessation [39], while stopping smoking, even after middle age, avoids much of the risk of lung cancer attributable to tobacco [40]. Our study illustrates the association between life expectancy, mortality trends and their respective position in the global smoking epidemic model [41, 42]. For men, increases in life expectancy across many age groups have been seen largely because of declines in lung cancer mortality resulting from effective measures of tobacco control over the last decades. At the same time, our results point to the very limited gains in life expectancy among women in several European countries that often goes against the narrative of the east vs. west divide and the reduction of gender inequalities in cancer mortality. These result from sex-specific historical trends in tobacco consumption [43, 44] and recent trends in female smoking prevalence [45, 46] suggesting that the global smoking epidemic model links to cultural and legislative divergence at the country level, including social acceptance and the targeting of women by the tobacco industry [47]. According to our study, the losses in age-specific life expectancy are minimal in Denmark, Ireland, the Netherlands, and the U.K. relative to the marked impact seen among middle-aged women in France, Spain and several countries in Central and Eastern Europe (Austria, Hungary, Slovenia and Croatia). The smoking-attributable mortality (for which lung cancer predominates) peaked in different periods in different European regions, with forecasts indicating a decline in smoking-attributable mortality occurring before 2030, that will continue until 2040 and will be halved by 2065 [48]. Evidently, the large variations in European regions and countries reflect the relative aggressivity of tobacco control policy, which determines to a considerable extent the potential of primary prevention [49].

After lung, cancers of the breast, colorectum and prostate exhibit the highest mortality rates in Europe [50]. Our analysis provides an illustration of progress in cancer control through rate reductions in breast and prostate cancer mortality that are related to a broad and complex spectrum of cancer control interventions, including early detection through mammography [51], PSA testing and curative treatment [52], as well as improved diagnosis and better access to effective and breakthrough treatments [53]. In contrast, our results show that the respective gains in life expectancy related to these cancers is more limited in Eastern Europe. The gains in LE due to declines in colorectal mortality do not however show clear East-West differences; these may reflect in part changes in the prevalence and distribution of key risk factors that affect incidence as well as the extent to which population-based colorectal screening has been fully implemented [54].

A key strength of our study is the comprehensive assessment of LE changes in relation to CVD and cancer mortality trends that highlights inequalities between and within EU countries by sex and age. A limitation is the use of cause of death statistics, which varies in its robustness from country to country and represents an oversimplification, given for example, smoking is a ubiquitous risk factor that could have a determining role in the development of both CVD and cancer [38] leading to the presence of competing risks in terms of the underlying cause of death. Another caveat is the prospect of multi-morbid chronic conditions which may overlap [55], especially at older ages. Some studies have reported an association with certain cancer therapies and increased risk of CVD [56], including breast, prostate, and bladder cancer patients [57]. A final limitation stems from the methodology used here, which does not provide insights into possible year-to-year fluctuations or trends from 1995–2019 that are masked by this comparison of the first and last five-year periods only.

At present, CVD and cancer rank as either the first or second leading causes of premature death in the majority of countries worldwide, notably in those with higher human development levels and robust health systems and infrastructure [58, 59]. While the increasing global dominance of chronic diseases is evident from the underlying mortality statistics, the extent of local epidemiologic transition depends on national trajectories of social and economic development [60]. This study provides a timely, in-depth analysis of gains in life expectancy over the last quarter-century that are attributable to progress in the control of CVD versus common cancer types; it underscores the continued existence of an East-West divide in life expectancy across the EU27 + 1, evident on benchmarking the founding EU vs. A10 countries.

### Electronic supplementary material

Below is the link to the electronic supplementary material.

Supplementary Material 1
