# Supplementary material for: Gains in life expectancy from decreasing cardiovascular disease and cancer mortality – an analysis of 28 European countries 1995–2019
Source: Eur J Epidemiol. 2023 Sep 7;38(11):1141–52. doi: 10.1007/s10654-023-01039-8 (PMC10663201; doi:10.1007/s10654-023-01039-8)
Supplement: Supplementary file 1 — Supplementary Material 1 [file 10654_2023_1039_MOESM1_ESM.docx]

**Appendices**

**A**

Figures were not calculated in the cases of Romania Malta and Cyprus for which life tables were missing from the HMD. For these countries 5-year mean average LEs at birth were calculated from the Eurostat database for the summary LE change diagrams. Life tables for France and the U.K. were only available for the periods 1995-1999 vs. 2010-2014, and in Croatia for 2000-2004 vs. 2015-2019. To ensure the comparability of cause-specific mortality in smaller populations over time five-year average ratios were calculated for every country by sex for the above causes of death for available periods of the life tables. Other than for Slovakia and Latvia where cause of death data was missing for 1995, 1996-1999 data were averaged and compared to 1995-1999 life tables. In Portugal, cause of death data was missing for 2019, hence data for 2015-2018 were averaged and compared to the 2015-2019 life tables. Finally, in Germany cause of death figures and life tables were available for 2005-2009 vs. 2010-2014 for larynx, lung, bronchus trachea, brain and central nervous system, thyroid, Hodgkin lymphoma, and leukaemia; 1995-1999 vs. 2010-2014 were used for other causes of cancer deaths.

**B**

**Figure: Top and bottom performers in contributions to increasing life expectancy in terms of cancer as a cause of death in the EU between 1995-1999 and 2015-2019**

A - males

Data source: HMD, Eurostat database

B - females

 Data source: HMD, Eurostat database
